# SUPPORT Tools for evidence-informed health Policymaking (STP) 16: Using research evidence in balancing the pros and cons of policies

**DOI:** 10.1186/1478-4505-7-S1-S16

**Published:** 2009-12-16

**Authors:** Andrew D Oxman, John N Lavis, Atle Fretheim, Simon Lewin

**Affiliations:** 1 Norwegian Knowledge Centre for the Health Services, P.O. Box 7004, St. Olavs plass, N-0130 Oslo, Norway; 2 Centre for Health Economics and Policy Analysis, Department of Clinical Epidemiology and Biostatistics, and Department of Political Science, McMaster University, 1200 Main St. West, HSC-2D3, Hamilton, ON, Canada, L8N 3Z5; 3 Norwegian Knowledge Centre for the Health Services, P.O. Box 7004, St. Olavs plass, N-0130 Oslo, Norway; Section for International Health, Institute of General Practice and Community Medicine, Faculty of Medicine, University of Oslo, Norway; 4 Norwegian Knowledge Centre for the Health Services, P.O. Box 7004, St. Olavs plass, N-0130 Oslo, Norway; Health Systems Research Unit, Medical Research Council of South Africa

## Abstract

*This article is part of a series written for people responsible for making decisions about health policies and programmes and for those who support these decision makers*.

In this article, we address the use of evidence to inform judgements about the balance between the pros and cons of policy and programme options. We suggest five questions that can be considered when making these judgements. These are: 1. What are the options that are being compared? 2. What are the most important potential outcomes of the options being compared? 3. What is the best estimate of the impact of the options being compared for each important outcome? 4. How confident can policymakers and others be in the estimated impacts? 5. Is a formal economic model likely to facilitate decision making?

## About STP

*This article is part of a series written for people responsible for making decisions about health policies and programmes and for those who support these decision makers. The series is intended to help such people ensure that their decisions are well informed by the best available research evidence. The SUPPORT tools and the ways in which they can be used are described in more detail in the Introduction to this series *[1]. *A glossary for the entire series is attached to each article (see Additional File *1*). Links to Spanish, Portuguese, French and Chinese translations of this series can be found on the SUPPORT website *http://www.support-collaboration.org. *Feedback about how to improve the tools in this series is welcome and should be sent to*: STP@nokc.no.

## Scenario

*You work in the Ministry of Health. The Minister of Health has asked you to present a summary of the expected benefits, harms and costs of an important change in health policy that is being considered*.

## Background

In this article, we suggest five questions that policymakers and those who support them can ask when considering how to ensure that judgements about the pros and cons of health policy and programme options are well-informed by research evidence. Such questions can be asked, for instance, in scenarios, such as the one described above.

**Figure 1:**
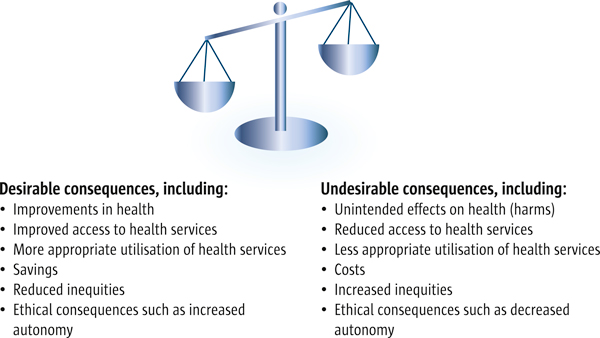
**Balancing the pros and cons of health policies and programmes**. Decisions about health policy or programme options require judgements about whether the desirable consequences of an option are worth the undesirable consequences

Research alone does not make decisions [2]. Judgements are always required, including judgements about what evidence to use, how to interpret that evidence, and our confidence in the evidence. More importantly, decisions about options require judgements about whether the anticipated desirable consequences outweigh the undesirable consequences (see Figure 1) [3]. In addition to making judgements about how big the impacts are likely to be, decision-making processes require judgements about how important the impacts are, the resources that are required to implement the option [4], and the extent to which the option is a priority relative to other ways in which those resources might be used.

It would be simple to make a decision if an option was expected to have large benefits with few downsides and little cost, if we were confident about the evidence and the importance of the benefits, and if the option was a clear priority. Unfortunately, this is rarely the case. More often the expected impacts and costs are uncertain, and complex and difficult judgements must be made.

The questions we propose here do not reduce the need for judgements. However, more systematic considerations and discussions of these questions could help to ensure that important considerations are not overlooked and that judgements are well informed. These could also help to resolve disagreements or at least help to provide clarification. If these judgements are made transparently they could help others to understand the reasoning behind health policy decisions.

Preparing and using a balance sheet (as explained in Table 1 and addressed in the first four questions discussed below) can facilitate well-informed decision making. Sometimes using a formal economic model, such as a cost-effectiveness analysis, can also be helpful. This latter issue is addressed in the fifth question discussed in this article. The considerations we suggest here are based on the work of the GRADE Working Group [5]. Although the Group's focus has been primarily on clinical practice guidelines, their approach to decisions about clinical interventions can also be applied to policies and programmes [6].

**Table 1:** The pros and cons of balance sheets

A balance sheet is a simple but powerful way to present the advantages and disadvantages of different options, including policy options [17,23]. In this section we describe the evidence and judgements needed to prepare and use a balance sheet such as the one shown in Table 2. We also describe the advantages of using a balance sheet compared to the use of non-systematic and non-transparent judgements of experts.
The aim of a balance sheet is to help decision makers develop an accurate understanding of the important consequences of the options being compared. Balance sheets help to achieve this in a number of ways. Firstly, they condense the most important information, thus enabling efficient consideration. Secondly, balance sheets focus attention on the most important outcomes. This increases the likelihood that decision makers will gain an accurate perception of what is known about the impacts of the options being considered and the important consequences. Thirdly, the act of constructing a balance sheet is a helpful mechanism for organising thinking, structuring evidence analysis, and focusing debate. Fourthly, balance sheets can help to develop more explicit judgements about what the most important consequences of policy options are, the underlying evidence, and subsequent judgements about the balance between the relative advantages and disadvantages of the various options. Lastly, balance sheets can provide other decision makers with 'raw information', thereby helping them to apply their own judgements about the trade-offs between desirable and undesirable consequences.
But two important limitations also need to be considered when using balance sheets in decision making. Firstly, when there are complicated trade-offs between multiple outcomes, judgements may require a high level of information processing by policymakers. Secondly, when weighing up different outcomes, the value judgements employed by policymakers could remain implicit. Formal economic modelling may help to address these limitations by making any underlying assumptions (including value judgements) more explicit. This enables the use of sensitivity analyses to explore the effects of both uncertainties and varying assumptions on the results.

## Questions to consider

The following five questions can be used to guide the use of evidence to inform judgements about the pros and cons of health policy and programme options:

1. What are the options that are being compared?

2. What are the most important potential outcomes of the options being compared?

3. What is the best estimate of the impact of the options being compared for each important outcome?

4. How confident can policymakers and others be in the estimated impacts?

5. Is a formal economic model likely to facilitate decision making?

The first four questions are intended to guide the use of balance sheets in policy decision making. Answering the final question can help to ensure that the scarce resources used in full economic analyses are applied where they are needed most.

Ideally, balance sheets (and economic models) should be constructed by researchers or technical support staff together with policymakers. They should also be based on systematic reviews for the same reasons described elsewhere that highlight the importance of systematic reviews in general [7]. We will not consider the many detailed judgements that must be made when constructing a balance sheet as these have been addressed elsewhere [8]. Policymakers are rarely, if ever, in a position where they are required to make all such judgements themselves. Yet even in instances where there is competent technical support to prepare a balance sheet, it is important that policymakers know what to look for and what questions to ask. This ensures that balance sheets can be used judiciously to inform the decisions for which policymakers are accountable.

### 1. What are the options that are being compared?

When using a balance sheet such as the one shown in Table 2, the first consideration is the need to identify what options are being compared. Often this is not as straightforward as it sounds (see Table 3, for example). Those preparing a balance sheet must decide on both the option being considered *and *the comparative option. Typically, the comparison is the status quo. However, the status quo is likely to vary from setting to setting. Decisions need to be made, therefore, about which characteristics of the status quo are:

**Table 2:** Should the licensing of tobacco retailers be conditional on not selling tobacco to minors?

**Population**: Minors (as defined by a legal age limit) **Setting**: Europe **Interventions**: Licensing of tobacco retailers + compliance checks* Comparison: No licensing or compliance checks					
		**Impact**			
	**Pessimistic**	**Best guess**	**Optimistic**	**Number of studies**	**Quality of the Evidence (GRADE)**^‡^

**Reduced number of smokers per year**	0	?	1,650 in the country (population 4.5 million)	4	Very low^‡^

**Life years saved per year**	0	?	9,240 in the country (population 4.5 million)	4	Very low^§^

**Cost per year**	€10,5 million (3 controls per year)	?	€2 million (1 control per year + internal control)	0	Very low**

*The proposed licensing law in the European country in question would require retailers to have a licence to sell tobacco. The policy options that were considered included three compliance checks per year, and one per year together with internal control. Compliance checks (by a teenager attempting to purchase tobacco) are done to ensure that tobacco is not being sold to minors. The penalty for non-compliance is the loss of a retail licence. Internal control requires the retailers themselves to have routines for controlling the sale of tobacco to minors

^†^See Table 8

^‡^The systematic review used as a basis for this summary (which was not used in the expert report to which we refer in subsequent tables) included one relevant randomised trial and three controlled before-after studies with important limitations. There was a high risk of bias for the estimated impacts on smoking prevalence. Important inconsistencies in the results lacked a compelling explanation. The studies in the review were based in the United States (2), the United Kingdom (1) and Australia (1), with differences in the interventions and uncertainty about whether similar results would be expected in the country where this policy was being considered. Two studies found an effect in lower age groups that was not sustained in one study; two studies did not find a change in smoking behaviour. It is difficult to estimate, based on these studies, what the best estimate would be of the impact of licensing of tobacco retailers with compliance checks on reducing the number of people who smoke. A lower estimate would be that there would be no impact from this intervention. The upper estimate is taken from an expert report (see Tables 3 5)

^§^The upper estimate of life years saved, which is taken from the same expert report, has the same limitations as the estimate of the impact on smoking behaviour, since it is based on that estimate. In addition, it is based on assumptions about what would happen long beyond the length of the studies that had evaluated impacts on smoking behaviour as well, and assumptions about the impact of the changes in smoking behaviour on mortality

**The estimates of the cost of the policy are taken from the expert report (described in subsequent tables in this article). These are based on an estimate of how many retailers sold tobacco, an assumption about what it would cost to process each licence, and an assumption about the costs of each compliance check

**Table 3:** What is being compared? Case example: The licensing of tobacco retailers

The reduction of teenage smoking was a priority for a Minister of Health in a European country. A report of policy options to achieve this was commissioned by the government concerned and a report was prepared by leading public health experts. One of the policy options considered in the report was the licensing of tobacco retailers. The loss of such a license was proposed as a penalty for the illegal selling of tobacco to minors. This option was compared in the report to the status quo, namely the absence of licensing for tobacco retailers. The public health experts did not undertake or use a systematic review, nor did they specify which characteristics of the policy option (or comparator) they considered to be crucial or important.
A number of important issues were not considered in the report. Important differences, for example, might have existed between the status quo of the areas where the policymakers considered implementing the policy and those where the studies were done. Such considerations may have included other policies already in place to reduce the sales of tobacco to minors. It is possible that existing legislation may already have made the sale of tobacco to minors illegal, or contained other methods by which legislation could be enforced (e.g. through fines or other penalties for the illegal sale of tobacco, face-to-face education of retailers (informing them about the legal requirements), or media campaigns (to raise community awareness). There might also have been differences in the ease with which minors could obtain tobacco from other sources (e.g. from parents and friends or through theft).
The experts explicitly considered two policy options for the licensing of tobacco retailers, namely three compliance checks per year (by a teenager attempting to purchase tobacco) to make sure that retailers were not selling tobacco to minors, and one compliance check per year together with internal control (requiring retailers themselves to control that tobacco is not being sold to minors). The penalty for non-compliance in both cases was the loss of the relevant licence. Other ways of enforcing licensing are possible, some of which have been evaluated in other studies. The experts writing this report did not explicitly address whether differences in approaches to licensing enforcement were likely to result in important differences in the effectiveness of the policy.

• Crucial - such that research with a comparison without those same characteristics would be excluded

• Important but not crucial - such that research with a comparison without those same characteristics would be included, but with less confidence that the results would be the same in the chosen setting, and

• Unimportant - such that we would be confident that the results are likely be the same in the chosen setting

These same judgements also need to be made about the options being considered: which of their characteristics are crucial, important or unimportant in terms of affecting the likely impacts?

### 2. What are the most important potential outcomes of the options being compared?

Policymakers, in general, are motivated by the desire to serve the people they represent and should be interested primarily in the impacts of policy and programme options on outcomes that are important to those affected (see, for example, Table 4). These include health outcomes, access to - or utilisation of - health services, unintended effects (harms), and resource use (costs or savings) (see Figure 1). Other often important consequences include the distribution and equity of benefits and costs [9], and spillover effects to other sectors. Ethical consequences such as those related to a reduction in people's autonomy, may also be important.

**Table 4:** What are the most important outcomes? Case example: The licensing of tobacco retailers

The primary outcome considered by the expert report commissioned by the government concerned was the prevalence of smoking. This was recognised to be a surrogate outcome for the consequences of smoking. The impact on life years saved was estimated based on the estimated impact on the prevalence of smoking and on epidemiological data linking smoking to mortality. Impacts on morbidity were not considered. Other impacts that were explicitly considered by the experts were administrative costs, political acceptability and public acceptability. There are a number of other outcomes that the expert report could have considered, including:
• Costs to retailers and potential harms (e.g. increased theft or cross-border shopping)
• Who would pay the administrative costs of such schemes
• The potential differences in the impacts of the policy on different populations (e.g. socio-economically disadvantaged minors or those living close to the country's border (who could potentially cross over into a neighbouring country to purchase tobacco)
• Ethical consequences (e.g. those related to the use of a minor or person pretending to be a minor for compliance checks, or the fairness of the policy in relation to the potentially different impacts on different groups of minors and different retailers)

Being explicit about which outcomes are important can help to ensure that the important consequences of an option are not overlooked. It can also help to ensure that unimportant consequences are not given undue weight. This is particularly important for surrogate outcomes - i.e. outcomes that are not important in and of themselves. They are considered important because they are believed to reflect important outcomes. For example, people do not typically regard their blood pressure as an important concern. What makes the issue of blood pressure important is its association with strokes, heart attacks and death, all of which *are *very much of importance to people. So when considering options targeted at hypertension (or other cardiovascular risk factors), decisions should be based on the impacts of these options on important outcomes (cardiovascular disease). Evidence of impacts on blood pressure alone is only a form of indirect evidence of the impacts on cardiovascular disease.

### 3. What is the best estimate of the impact of the options being compared for each important outcome?

Deciding whether the desirable impacts of an option are worth the undesirable impacts requires an estimate of how large these different impacts (and their economic consequences) will be. Ideally, this should take the form of a comparison between what could be expected for every important outcome if an option *were *to be implemented, and what could be expected if it *were not *- or what could be expected if a different option were implemented instead (see Table 5, for example). It is also useful to know how precise each estimate is - i.e. what the 'confidence interval' is for each estimate (this is explained further in Table 6).

**Table 5:** What are the best estimates of the impacts? Case example: The licensing of tobacco retailers

The expert report on policies to reduce teenage smoking commissioned by the government concerned estimated that licensing tobacco retailers would result in a 10% relative reduction in the number of smokers. Using the current prevalence of smokers as a reference, the absolute effect of the policy was estimated to be a reduction of 1,650 smokers per year. Based on epidemiological models of the increased risk of dying due to smoking, the experts estimated that this policy would save 9,240 lives per year. No confidence intervals were provided, although it was noted that the actual effect was very uncertain and a range of estimates was used to calculate the cost-effectiveness of licensing tobacco retailers. Administrative costs were estimated, based on an estimate of how many retailers sold tobacco, an assumption about what it would cost to process each licence, and an assumption about what each inspection would cost (to check compliance with the requirement not to sell tobacco to minors).
Using these different assumptions, the total estimated cost was between €7.2 million and €10.5 million per year.

**Table 6:** Confidence intervals

A confidence interval (CI) is the range around an estimate which conveys how precise the estimate is. The confidence interval is a guide that represents how sure it is possible to be about the quantity we are interested in (e.g. the effect of a policy option on an outcome of interest). The narrower the range between the upper and lower numbers of the confidence interval the more precise the estimate is and the more confident it is possible to be about the true value. The wider the range the less certain it is possible to be. The width or range of the confidence interval reflects the extent to which chance may be responsible for an observed estimate (wider intervals reflect the greater likelihood of chance being a factor). A 95% CI means that we can be 95% confident that the true size of an effect is between the lower and upper confidence limit. Conversely there is a 5% chance that the true effect is outside this range.

It is important that decision makers recognise the difference between estimates of effect that are presented as *relative *effects, and those that are presented as *absolute *effects. Patients, health professionals, and people making decisions about health policies and programmes are more likely to decide to use an intervention if its effects are reported as a relative effect than if they are reported as an absolute effect [10]. For example, a study reported that 61% of a sample of health professionals in Australia agreed to implement a colorectal cancer screening programme that would reduce the rate of deaths from bowel cancer by 17% (the relative risk reduction). In comparison, only 24% of the health professionals agreed to implement a programme that produced an absolute reduction in deaths from bowel cancer of 0.4% (the absolute risk reduction) [11]. Both estimates were, in fact, from the same programme (for an explanation of the difference between relative and absolute effects see Table 4 in Article 10 of this series [9]).

### 4. How confident can policymakers and others be in the estimated impacts?

Six factors can lower our confidence in estimates of the impacts of a policy or programme [12]:

• A weak study design

• Other study limitations

• Imprecision

• Inconsistent results

• Indirectness of the evidence

• Publication bias

An assessment of these factors is inevitably technical. Policymakers do not need to have a detailed understanding of these factors or how they are assessed. But both policymakers and their technical support staff can still benefit from understanding why it is important to consider these factors.

Studies in which a programme is randomly assigned reduce the risk of unknown or unmeasured differences between the groups being compared. This gives greater confidence that impacts are attributable to the programme and not some other factor [13-15]. Study designs that do not use random assignment can account only for differences that are measured. For example, a study in which communities are randomly assigned to a programme or policy option, such as the licensing of tobacco retailers, would provide more compelling evidence of the impacts of an option than a study would if it compared communities that had decided themselves whether to implement a particular option. This is because communities that decide to implement an option are likely to differ from those that do not in ways that could have an impact on the outcomes of interest (in this case, smoking prevalence). It would therefore be impossible to know whether the differences in outcomes were due to the policy or programme option or due to those other differences between the communities.

Other study limitations can affect both randomised and non-randomised impact evaluations. Incomplete data or the unreliable measurement of outcomes, for instance, may increase the risk of an estimate being biased, and therefore lower confidence in the derived estimates.

Imprecision (as indicated by a wide confidence interval) also lowers the confidence with which chance can be ruled out as a factor shaping any observed differences in outcomes between compared groups, and consequently our confidence in an estimated effect. (Table 6 explains the concept of confidence intervals in further detail)

If different studies of the same policy or programme option have inconsistent results and there is no compelling explanation for such differences, there will also be less confidence in knowing the expected impacts arising from implementing the option.

There are several ways in which studies might not be directly relevant to a particular question, and therefore result in less confidence in the results. As noted above, if an indirectly relevant outcome (such as blood pressure) is measured in place of an important outcome (cardiovascular disease), there will be less confidence in the impacts on the important outcome (for which the indirect outcome is a surrogate). If only *indirect *comparisons are provided, confidence will also be lower. We would be less confident in studies of an option that lacked head-to-head comparisons, for example, between the option compared to a control (with no intervention) and studies of a different option compared to a control. Other ways in which evidence can be indirect include differences between a study and the setting of interest in:

• The characteristics of the population

• The option being considered, or

• The status quo or comparison option

Studies that find statistically significant effects are often more likely to be published than those that do not [16]. When such 'publication bias' appears likely, confidence in estimates from published studies alone may also be lowered. Publication bias should be considered in instances where there are a number of small studies, especially if these are industry-sponsored, or if the investigators are known to share other similar conflicts of interest.

In summary, assessments of the 'quality' or robustness of evidence, and confidence in estimates of the likely impacts of options, depend on a consideration of *all *of the factors noted above. Although there are no fixed rules for assessing these factors, judgements related to the quality of evidence that explicitly address each factor help to reduce the likelihood of important factors being overlooked. They also help to reduce the probability of biased assessments of the evidence (see Table 7, for example). Using a systematic and transparent approach, such as the GRADE approach (see Table 8), makes it easier to inspect the judgements made [5].

**Table 7:** How confident are we in the estimated impacts? Case example: The licensing of tobacco retailers

The expert report commissioned by the government concerned concluded that the empirical basis for the licensing of tobacco retailers was "robust" but the basis for this judgement was unclear. The experts did not conduct, or cite, the systematic review that is referenced in Table 3, or any other systematic review as the basis for their estimates, even though a systematic review was available [24]. In contrast to the experts' unexplained judgement, an assessment of the evidence summarised in the systematic review using the GRADE approach, suggests that the quality of the evidence was very low for all the important outcomes (see Table 8 for further information related to the GRADE assessment system). Table 1 summarises the findings of the experts' report in the form of a balance sheet for this policy decision and shows an assessment of the quality of the evidence for the three estimates using the GRADE approach.
The authors of the systematic review (which included a broader range of interventions and study designs) concluded: "Interventions with retailers can lead to large decreases in the number of outlets selling tobacco to youths. However, few of the communities studied in this review achieved sustained levels of high compliance. This may explain why there is limited evidence for an effect of the intervention on youth perceptions about ease of access to tobacco, and on smoking behaviour." The 'pessimistic' estimates of the benefits in Table 1 are consistent with the findings of the systematic review and were not considered in the expert report.

**Table 8:** The GRADE system for assessing the quality of evidence

Evaluating the quality of evidence requires judgements about the extent to which one can be confident that an estimate of effect is correct. GRADE provides a systematic and transparent approach to making these judgements for each outcome important to a decision [12]. The judgements are based on the type of study design (randomised trials versus observational studies), the risk of bias (study limitations), the consistency of the results across studies, and the precision of the overall estimate across studies. Based on these considerations for each outcome, the quality of the evidence is rated as high, moderate, low, or very low, using the following definitions:	
**High**	Confident that the true effect lies close to that of the estimate of the effect
**Moderate**	The true effect is likely to be close to the estimate of the effect, but there is a possibility that it is substantially different
**Low**	The true effect may be substantially different from the estimate of the effect
**Very low**	Very uncertain about the estimate

### 5. Is a formal economic model likely to facilitate decision making?

Formal economic models, such as cost-effectiveness analyses and cost-utility analyses, can help to inform judgements about the balance between the desirable and undesirable consequences of an option [17]. Economic models can be valuable for complex decision making and for testing how sensitive a decision is to key estimates or assumptions. A model, though, is only as good as the data on which it is based. When estimates of benefits, harms or resource use come from low-quality evidence, the results will necessarily be highly speculative (an example is provided in Table 9).

**Table 9:** Is a formal economic model likely to help? Case example: The licensing of tobacco retailers

The expert report commissioned by the government concerned included an economic analysis. This concluded that the cost per life year saved by licensing tobacco retailers and conducting compliance checks was between approximately €900 and €92 000 with a best estimate of €8 000. The authors noted that there was substantial uncertainty about their estimates and suggested focusing on the range of estimates rather than the best estimate. Nevertheless they reported exact estimates (based on the assumptions they made) and concluded that the empirical basis for recommending licensing tobacco retailers was robust. As a result policymakers who failed to read this report critically could conclude (wrongly in our opinion) that the report provided high-quality evidence that the licensing of tobacco retailers was as cost-effective as (or more cost-effective than) a wide range of clinical preventive services paid for by the government. A more systematic review of the underlying evidence [24] and a summary of the findings that included more systematic and transparent judgements of the quality of the evidence (as shown in Table 1) would have provided a better basis for decision making.

A full economic model is more likely to help to inform a decision when there is:

• A large difference in the resources consumed between the compared options

• Large capital investments are required, such as the construction of new facilities

• Uncertainty about whether the net benefits are worth the incremental costs

• Good quality evidence regarding resource consumption

An economic model can also be used to clarify information needs by exploring the sensitivity of an analysis to a range of plausible estimates.

Unfortunately, published cost-effectiveness analyses, particularly those undertaken for drugs, have a high probability of being flawed or biased. They are also specific to a particular setting which may differ in important ways from the setting of interest [18]. Policymakers may thus consider developing their own formal economic models. To do this, they must have the necessary expertise and resources.

## Conclusion

Policy decisions are informed by assessments of the balance between the pros and cons of options. As we have recommended, these should be done systematically and transparently. When the net benefit (i.e. the difference between the desirable and undesirable consequences) is large in relation to the costs, we are more confident about a decision. When the net benefit is small in relation to the costs, we are less confident.

Generally, the less confident we are about the likely impacts of an option, the less confident we will be when deciding what to do. There are exceptions to this: firstly, we may have so little confidence about the impacts of something that it is easy to decide not to do it.

Secondly, even if there is little confidence in the benefits of a particular option it may be easy to decide to do something simply because there is little or no risk of harm, it doesn't cost much, and it might do some good. Many types of health information could be categorised as such. Policymakers, though, should be cautious about assuming that seemingly harmless polices and programmes *cannot *do harm [19]. Even something as simple as providing health information can, in fact, be deadly [20]. This is demonstrated by the advice given to mothers in many countries for nearly 50 years, that babies should sleep on their front. The seemingly harmless advice caused tens of thousands of deaths from sudden infant death syndrome [21].

Finally, despite important uncertainty about the likely impacts of a policy or programme, it may be easy to come to a decision that something that is promising should only be done in the context of a well-designed evaluation of its impacts [22].

Even when we are confident about the impacts of a policy or programme, it may not be a priority to implement it. The extent to which we are confident is a critical factor for deciding on what to do and the extent to which doing something is a priority. Other additional factors (such as those described in Table 10) may also determine whether policy or programme implementation is a priority or not.

**Table 10:** Factors that can determine the importance of implementing health policies and programmes

The following factors may sometimes be considered independently (or in combination) as criteria for setting priorities for implementing health policies and programmes:
• How serious the problem is -- the more serious a problem is, the more likely it is that a policy or programme that addresses the problem will be a priority
• The number of people that are affected by the problem -- the more people who are affected, the more likely it is that a policy or programme that addresses the problem will be a priority
• Benefits -- the larger the benefit, the more likely it is that a policy or programme will be a priority
• Adverse effects -- the greater the risk of undesirable effects, the less likely it is that a policy or programme will be a priority
• Resource use (costs) -- the greater the cost, the less likely it is that a policy or programme will be a priority
• Cost-effectiveness -- the lower the cost per unit of benefit, the more likely it is that a policy or programme will be a priority
• Impacts on equity -- policies or programmes that reduce inequities may be more of a priority than ones that do not (or ones that increase inequities)
Decisions about priorities should rest on shared criteria or reasoning such as the ideas shown above. They should also be open to inspection and they should be possible to appeal in light of considerations that stakeholders may raise. Regulation should ensure that these three conditions are met [25]. When criteria such as the above are used implicitly rather than explicitly, it is difficult to judge whether the criteria or the decisions were appropriate [26].

## Resources

### Useful documents and further reading

- Guyatt GH, Oxman AD, Vist GE, Kunz R, Falck-Ytter Y, Alonso-Coello P, Schunemann HJ, and the GRADE Working Group. GRADE: An emerging consensus on rating quality of evidence and strength of recommendations. BMJ 2008; 336:924-6.

- Guyatt GH, Oxman AD, Kunz R, Vist GE, Falck-Ytter Y, Schunemann HJ, and the GRADE Working Group. What is 'quality of evidence' and why is it important to clinicians? BMJ 2008; 336:995-8.

- Guyatt GH, Oxman AD, Kunz R, Jaeschke R, Helfand M, Vist GE, Schunemann HJ, and the GRADE Working Group. Incorporating considerations of resource use. BMJ 2008; 336:1170-3.

### Links to websites

- SUPPORT Summaries: http://www.support-collaboration.org/index.htm - Concise summaries of the pros and cons of health policies and programmes for low- and middle-income countries based on systematic reviews.

- GRADE Working Group: http://www.gradeworkinggroup.org - The Grading of Recommendations Assessment, Development and Evaluation (or GRADE) Working Group has developed an approach to grading the quality of evidence and the strength of healthcare recommendations.

## Competing interests

The authors declare that they have no competing interests.

## Authors' contributions

ADO prepared the first draft of this article. JNL, AF and SL contributed to drafting and revising it.

## Supplementary Material

Additional file 1 Glossary Click here for file
